# Design of the UWB Positioning System Simulator for LOS/NLOS Environments

**DOI:** 10.3390/s21144757

**Published:** 2021-07-12

**Authors:** Krzysztof Paszek, Damian Grzechca, Andreas Becker

**Affiliations:** 1 Department of Telecommunications and Teleinformatics, Faculty of Automatic Control, Electronics and Computer Science, Silesian University of Technology, Akademicka 16, 44-100 Gliwice, Poland; 2 Department of Electronics, Electrical Engineering and Microelectronics, Faculty of Automatic Control, Electronics and Computer Science, Silesian University of Technology, Akademicka 16, 44-100 Gliwice, Poland; Damian.Grzechca@polsl.pl; 3 Faculty of Information Technology, University of Applied Science and Arts, Sonnenstr. 96, 44139 Dortmund, Germany; Andreas.Becker@fh-dortmund.de

**Keywords:** UWB, sensor data analysis, simulator, data reconstruction

## Abstract

UWB is a rapidly developing technology characterised by high positioning accuracy, additional data transferability, and communication security. Low costs and energy demand makes it a system that meets the requirements of smart cities (e.g., smart mobility). The analysis of the positioning accuracy of moving objects requires a ground truth. For the UWB system, it should have an accuracy of the order of millimetres. The generated data can be used to minimize the cost and time needed to perform field tests. However, there is no UWB simulators which can consider the variable characteristics of operation along with distance to reflect the operation of real systems. This article presents a 2D UWB simulator for outdoor open-air areas with obstacles and a method of analysing data from the real UWB system under line-of-sight (LOS) and non-line-of-sight conditions. Data are recorded at predefined outdoor reference distances, and by fitting normal distributions to this data and modelling the impact of position changes the real UWB system can be simulated and it makes it possible to create virtual measurements for other locations. Furthermore, the presented method of describing the path using time-dependent equations and obstacles using a set of inequalities allows for reconstructing the real test scenario with moving tags with high accuracy.

## 1. Introduction

Internet of things (IoT) devices are ubiquitous and are presented in the industry and in houses where they provide a lot of information (i.e., allowing one to automate processes or take care about the safety or life of people). IoT devices are an indispensable element of smart cities or industry 4.0 (e.g., intelligent parking lots that provide information about available spaces and make it easier to find a previously parked car) [1]. Such devices are also used in sports or rehabilitation [2,3]. GPS and inertial navigation are widely applied to determine the position of an object [4,5]. However, in urbanized areas the GPS signal is often distorted or completely unavailable. Inertial navigation allows for positioning regardless of the infrastructure. However, the position error accumulates over the time [6]. Ultra-wideband (UWB) is a rapidly developing technology that has become a beneficial alternative/supplement to other systems used in the automotive industry due to its low energy demand, low costs, additional data transferability, and communication security [7,8,9,10,11]. The UWB technology enriches a range of network types that can be used in the IoT for smart city applications [12]. The UWB system, apart from the option of data transmission, allows for determining the position with high accuracy, which together with the growing interest in autonomous vehicles will be important in the smart cities or smart industries [13,14,15]. Positioning systems such as Lidar, radar, ultrasound, interaction sensors, or cameras provide information about the vehicle’s surroundings [16,17,18,19,20]. Their advantage lies in independence from infrastructure. However, they do not provide information about, for example, an obstacle located directly behind a sharp bend, and they do not inform in advance about a road collision or a traffic jam because they can only inform users about things they can see. To propagate information about dangers, vehicles must communicate with the environment (including other vehicles or infrastructure) [21]. The advantages of the UWB system, such as the aforementioned accurate positioning or the possibility to communicate, make this system widely researched [22,23,24]. For analysing the accuracy of localization algorithms (trilateration) or raw data processing methods, reference data (ground truth) is used to check their effectiveness and quality. Reference data can be obtained from any system, the accuracy of which should be an order of magnitude larger (lower error) than the accuracy of the analysed system. Another solution is to repeat a given test scenario many times while maintaining the same parameters. Then, the mean values from all approaches should be used as reference data. Due to the fact that the accuracy of the UWB system, with appropriate data analysis, is in the order of a few centimetres, the accuracy of the reference system applied has to be in the order of millimetres. In addition, the sampling frequency of the UWB system can reach 100 Hz, making it difficult to find a reference system. It seems that the best solution is to run a given test scenario multiple times and average the obtained results [25]. However, taking into account the measuring platform (and the whole test stand), which is a car or a remote-controlled model, it is almost impossible for a driver or a RC model operator to repeat the scenario many times while maintaining the same parameters.

Due to the UWB system’s accuracy, it is common to determine the accuracy of the whole system at reference points where the object stops or by passing over this type of point and reading the marker (e.g., RFID, video analysis) [4,26]. In this approach, there is no available information on the accuracy of the position at each point on the path that the object has followed (where UWB position data is available). Additionally, time synchronization (e.g., of video with UWB data) is challenging. Another way is to determine the accuracy by comparing the reference path (the assumed path), and the path plotted based on the UWB system data. On the other hand, the information about the time when a given position was acquired is omitted in this approach.

Taking into account the limitations of the reference system, it was decided that we build a simulator that would reflect the operation of the off-the-shelf (and currently applied) UWB system used for positioning objects in real conditions. The benefits of building the simulator do not only affect the quality of the prepared algorithms and the precise analysis of the results, but also save time that has to be spent on performing the test in a real test stand and allow to continue research in situations where access to the test stand is limited (e.g., due to weather conditions or limitations resulting from events such as the COVID19 pandemic or internal restrictions of a plant).

Positioning systems simulators (e.g., for Lidar, radar, IMU, etc.) are widely used for testing algorithms that analyse data derived from them [27,28]. Simulation data is easier to obtain and does not require time-consuming testing in the field. However, there is a lack of a UWB simulator that would consider the variable characteristic of operation along with distance.

The UWB positioning system provides distances between network nodes, namely tags associated with the positioned object and the anchors (reference points). Obtained distances are used to calculate the position in the trilateration process [29,30]. Furthermore, distances from at least three reference points are needed to determine the position on a plane (2D). Thus, it is necessary to have a simulator that provides the same data as the real system.

Due to the lack of complete information on the transmission parameters and configuration of data exchange between the nodes of the UWB systems, it is difficult to build a simulator starting from a low level (e.g., frame transmission in the network) [31]. For this reason, a real system has been treated as a black box, and its operation has been modelled based on the information the system generates (i.e., distances).

The presented simulator is designed to simulate the UWB system for outdoor (open-air) areas with obstacles. The outputs of the proposed simulator are the distances between the user-defined network nodes, which can be acquired in both LOS and NLOS conditions. NLOS conditions are possible to obtain by the option of the simulator to add obstacles to the test stand. Among the variety of assumptions, the following have to be taken at first: the object moves according to the planned scenario (in a local coordinate system) and data from all network nodes within the tag range has to be collected. The simulator’s input data is the functions (shape) of the road/path in uniformly accelerated motion in the time domain, which reflects the passage of the object for a given test scenario.

It was observed that each of the applied positioning systems (of different vendors) based on UWB technology (and more precisely on DW1000 modules [32]) are characterised by slightly different parameters such as:

- Error in the determined distance;
- Variation of the measurements;
- Maximum sampling frequency; or
- Maximum ranging distance.

**Figure 1:**
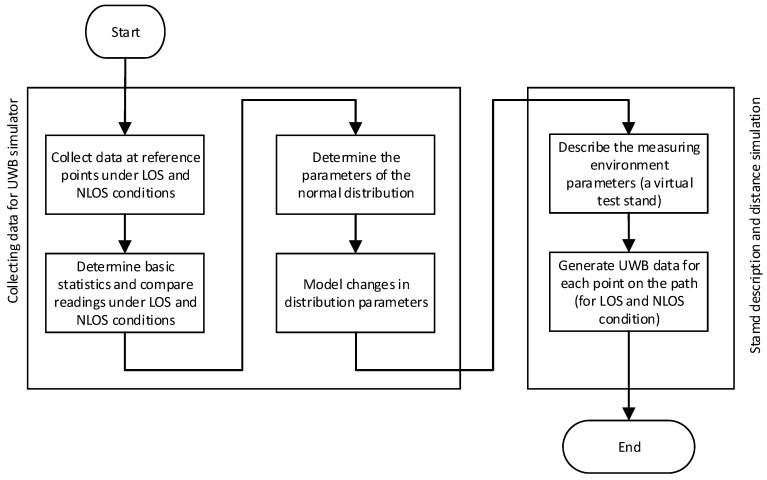
Main block diagram of the data analysis.

Data from the systems based on DWM1000 modules with an external omnidirectional antenna is used to build the simulator. The data was collected in an open area, with no additional objects close to the test stand. The influence of various materials (such as glass, people, wood) on distance measurement has been evaluated, and it was noticed that the metal plate has the greatest but reasonable influence on the signal. During the construction of the simulator, the worst-case scenario was assumed, which is a metal plate close to one node. The following stages of data analysis from the UWB system, building a virtual measurement environment along with collision detection, are presented in the diagram in Figure 1. The first stage of the analysis, the collection and analysis of data from the real system and its description, is discussed in Section 2. The creation of the virtual test stand, obstacle creation, and distance simulation are discussed in Section 3.

## 2. Collecting Data for UWB Simulator

**Figure 2:**
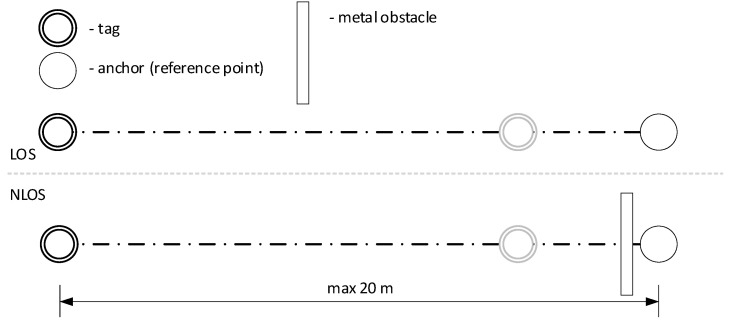
Test stand for static distance measurements (in both LOS and NLOS conditions).

Treating the positioning system as a black box, data of the measured distances between two nodes (tag and one anchor) was collected at the distances indicated in Table 1. The measurements were made in a stationary scenario (the nodes between which the measuring was performed were not moving during the ranging process) in two variants: line-of-sight (LOS) conditions and non-line-of-sight (NLOS) conditions (where the obstacle was simulated by covering one of the nodes with a 0.5 × 0.5 m metal plate—assuming the worst-case scenario) as presented in Figure 2. The test stand was located outside in an open area with no additional obstacle nearby. Each scenario includes 10,000 distance readings (rangings). The collected data and the designated statistics allow one to describe the operation of the system.

The system operation has been checked for both conditions separately (for LOS and NLOS conditions). The two-stage analysis of the collected data (distances) allows one to check whether the statistical parameters are similar and can be described with common parameters and show how much impact the obstacle has on the measurement of the distance between nodes using the UWB system.

**Figure 3:**
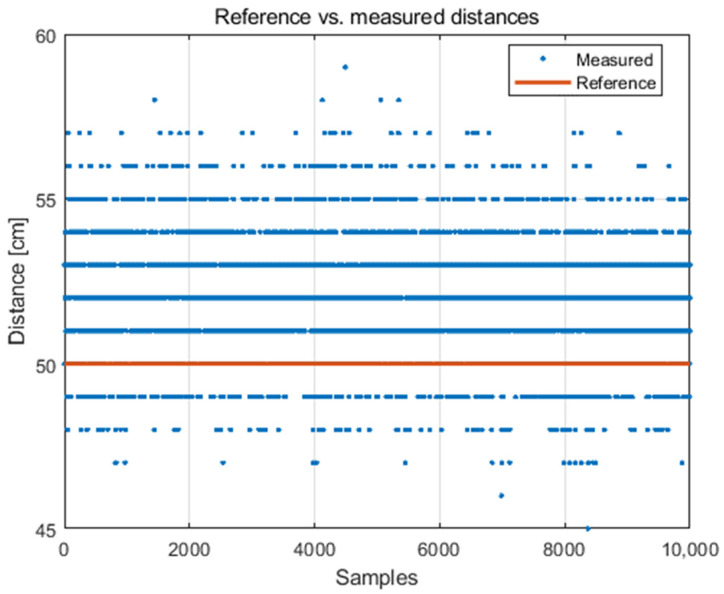
System readings (determined distances) for 50 cm distance between nodes.

In the first step, the data of the distance between nodes in LOS conditions was analysed. Sample system readings for a reference (fixed) distance of 50 cm between nodes are presented in Figure 3. It should be noted that the resolution of the system applied is 1 cm. The analysed system is characterised by an overestimation of the distance value for all the tested distances. There are many reasons for this phenomenon including the desynchronization of clocks between nodes, different accuracy of the clocks used, or the system configuration (delays). It is important to mention that this does not lay within the scope of the constructed simulator (the simulator is intended to reflect the real system, not to increase the accuracy of ranging).

**Figure 4:**
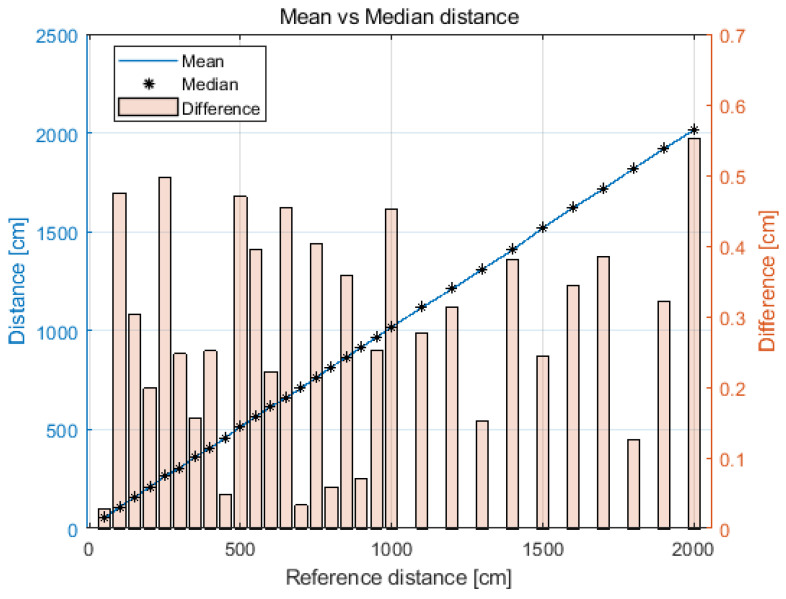
Difference between values of mean and median for each reference distance.

The collected statistical measures relating only to the distance measurement under LOS conditions are presented in Table 2. The mean values (d_mean_) are close to the median value (d_med_), which indicates a relatively symmetrical concentration of the measurements around the mean. The difference is presented in Figure 4. The maximum absolute value of the difference between the mean value and the median value (|d_mean_−d_med_|) is 0.6 cm (for the distance of 20 m).

To simulate the distances obtained from the real system precisely, the parameters of the data (distance) distribution have been determined to reflect the system’s operation. Such parameters allow one to add appropriate distortion to the exact data derived from the reference system (which in the discussed example are the accurate distances between two UWB nodes in the Cartesian coordinate system).

Even though the tests such as Lilliefors’ test and the Kolmogorov–Smirnov test reject the null hypothesis that the data comes from a normal distribution; based on the central limit theorem, the mean value of the random variable is similar to the normal distribution when the population is large enough. Therefore, having a sufficiently large sample, it is possible to approximate the distribution of the tested population to a normal distribution with a given value of mean and standard deviation.

**Figure 5:**
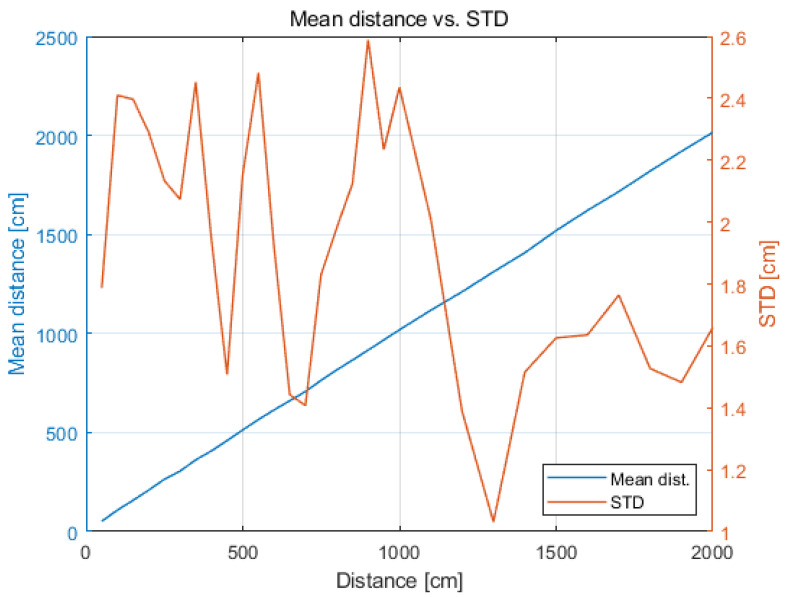
Change in standard deviation with distance.

Standard deviation changes over the predefined range (from minimum to maximum distances value) and is in the range of 1.0–2.6 cm, as shown in Figure 5.

With the reference value (which was determined using a laser rangefinder and a tape measure), it is possible to establish the examined UWB system’s accuracy. The essential statistical measurements of errors are summarised in Table 3.

**Figure 6:**
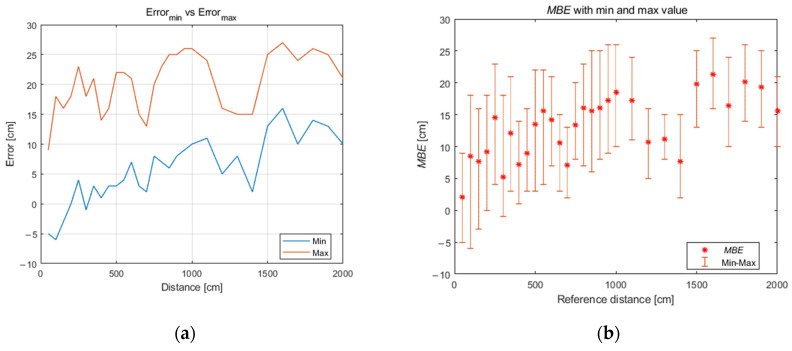
Distance error: (**a**) maximum and minimum value of error with distance; (**b**) range of distance error (MBE) for each reference distance.

The maximum (ε_max_) and minimum (ε_min_) distance error (1) varies with distance without a clear correlation with a reference value of distance which is indicated in Figure 6a. The mean error range for this set of data is 14.8 cm, the minimum value is 7 cm (for a reference distance of 13 m), and the maximum value is 24 cm (for a reference distance of 1 m; see Figure 6b):

(1) $$\varepsilon_{i}=d_{i}-\hat{d_{i}},$$

where $d_{i}$ is the measured distance and ${\hat{d}}_{i}$ is the reference distance.

**Figure 7:**
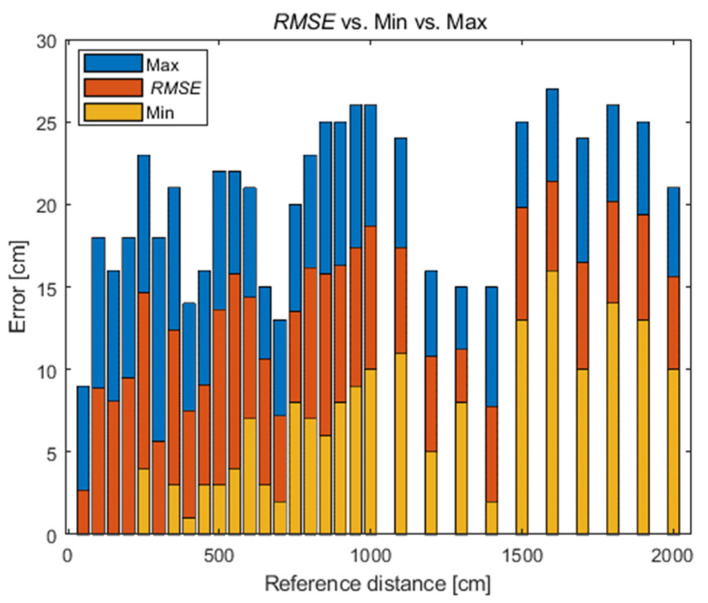
Change in RMSE with distance.

The fundamental accuracy measure in positioning systems (indoor and outdoor) is a root mean square error (RMSE, (2)). For the analysed system, the mean error changes with distance without being clearly monotonous. The average RMSE value for all reference distances is 13.3 cm, the maximum value is 21.4 cm (for the distance of 16 m), and the minimum value is 2.7 cm (for the distance of 50 cm). The other values are presented in Figure 7.

**Figure 8:**
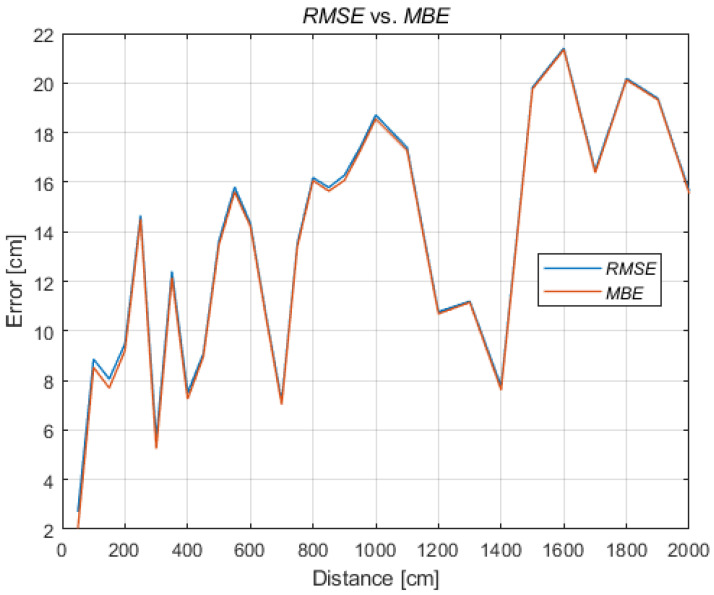
RMSE and MBE.

Due to the dominant overestimation of the distances, the mean bias error (MBE) (3) is close to the RMSE, presented in Figure 8 for all reference distances. However, when the system overestimates and underestimates the distances, the differences between the MBE and the RMSE increase. If the tested system is characterized by underestimation of the distance measurement, the MBE will be negative and will significantly deviate from the RMSE value. Therefore, the MBE value can be used to correct the distance in further data analysis during the position’s determination:

(2) $$RMSE=\sqrt{\frac{1}{N}\overset{N}{\underset{i=1}{\sum }}{\left(d_{i}-\hat{d_{i}}\right)}^{2}}$$

(3) $$MBE=\frac{1}{N}\sum_{i=1}^{N}\left(d_{i}-\hat{d_{i}}\right),$$

where N is the number of measured distances (in one reference distance), $d_{i}$ is the measured distance, and ${\hat{d}}_{i}$ is the reference distance. 

**Figure 9:**
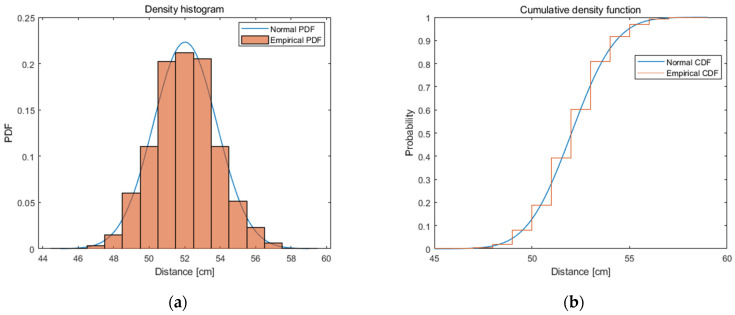
Example: (**a**) density histogram; and (**b**) CDF with distance.

The parameters (mean value and standard deviation) of the normal distribution on the test sample of 10,000 samples were determined for all reference distances. An example of the probability density function and cumulative distribution function of the normal distribution, as well as the empirical functions, are presented in Figure 9a,b, respectively. Thus, it can be said that the normal distribution largely approximates the distribution of the analysed system and allows for the reproduction of the system’s operation.

**Figure 10:**
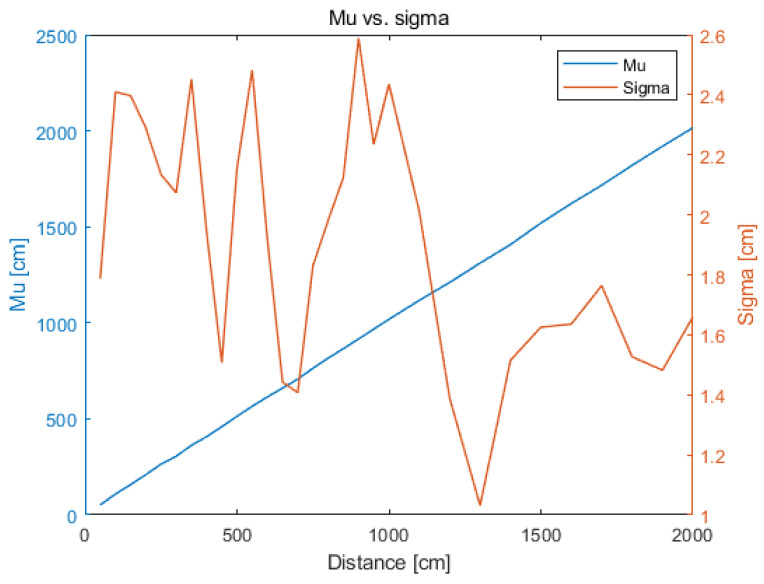
Normal distribution parameters.

The determined parameters of the distributions describe the data collected at 50 cm intervals (and for distances above 10 m at 100 cm intervals). Figure 10 shows the parameters of normal distributions fitted with the collected data at reference distances. To simulate the operation of the system at other distances, changes in distribution parameters can be modelled (approximated).

**Figure 11:**
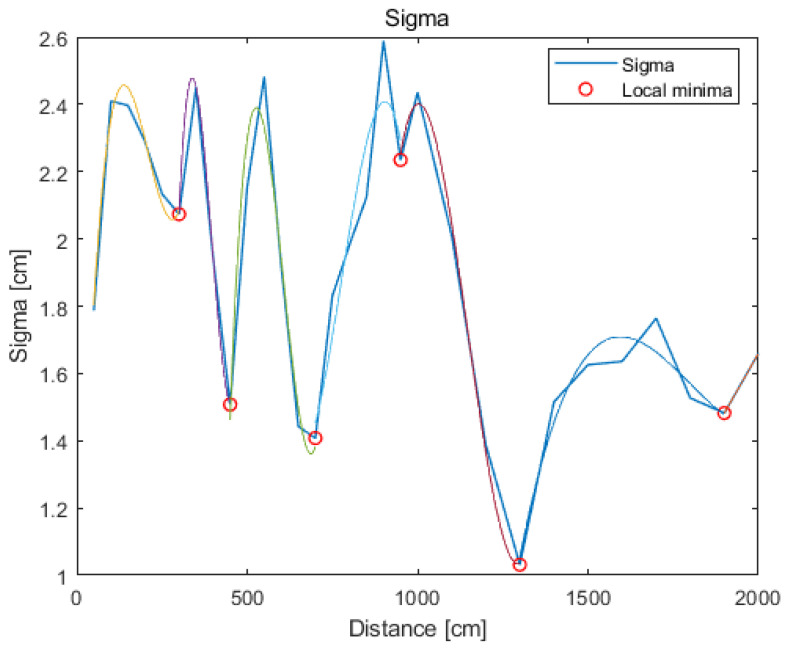
A polynomial approximation of the standard deviation.

The function which approximates the changes in standard deviation is divided into subranges, and for each subrange a polynomial is fitted. The following functions for approximation have been applied: 2nd and 3rd-degree polynomial, linear regression, power function. The best accuracy was obtained in subranges using the 3rd-degree polynomial. Fitting the functions (polynomials of at the most third degree) on a subrange allows one to model the changes at points where the reference measurement was not performed (between reference distances). For this purpose, local minima have been established along with the measurements range. The minimum number of measurement points (reference distances) between successive local minima is used as a parameter limiting the creation of an excessive number of approximating functions. The minimum number of measurement points is chosen empirically, and it consists of three points. In the next step, the polynomial is fitted at the intervals defined by the two successive local minima. In this way, the standard deviation changes as a function of distance is approximated (see Figure 11). This approach results in a good approximation of the systems’ accuracy between the reference points.

The above procedure has to be repeated for the second parameter of the normal distribution, namely the mean value. The function that approximates the mean in given distances is expected to be a linear function due to the continuous increase in the distance between the tag and the anchor at successive measurement points and, thus, reduced signal-to-noise ratio (SNR). However, it has to be kept in mind that the system error changes with the distance in an irregular manner (as shown in Figure 9). Therefore, to model the nonlinear changes in the system’s operation, a parameter other than the mean value of the distance must be found. The parameter that shows the distance error and can be applied to reproduce the mean value (based on accurate value) is the MBE. The analysed real system is characterised by overestimation of the distance. Therefore, the MBE is always positive.

**Figure 12:**
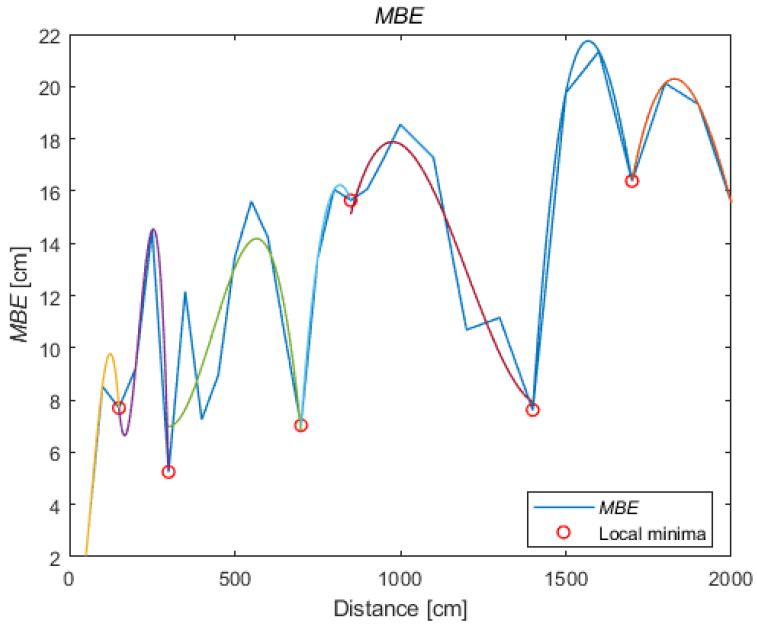
A polynomial approximation of the MBE.

In the same manner as for the standard deviation, to represent the MBE value in points between successive measurement points, the error change should be modelled. For this purpose, local minima were also determined with a minimum distance of three reference distances, and a polynomial of at the most third degree was approximated (see Figure 12).

The determined approximating functions and their domains allow for the reconstruction of the system operation at various distances in LOS conditions.

Similar data analysis has to be performed for measurements of distances under NLOS conditions. The collected statistical measures on the distance readings are summarised in Table 4. The influence of a metal obstacle on distance measurement has been reported. The mean value of the standard deviation (d_std_) for all measurements increased to 6.1 cm (by 217%), the minimum value increased to 1.15 cm (by 11%), and the maximum value increased to 18 cm (by 594%).

The positioning system error for NLOS conditions has also changed. In terms of the *RMSE*, there is an average increase to 35.1 cm (by 164%), the minimum value has decreased to 1.5 cm (by 43%), and the maximum error value has increased to 85.6 cm (by 300%). Exact values are listed in Table 5.

The metal obstacle (NLOS conditions) affects the real positioning system’s operation. The error of the distances determined by the real system is higher by an average of 164%. The normal distribution parameters (mean value and standard deviation) are calculated analogically to the procedure under LOS conditions to reflect the system’s operation under NLOS conditions. Then the changes in error and deviation as a function of distance are modelled.

## 3. Simulator Description

**Figure 13:**
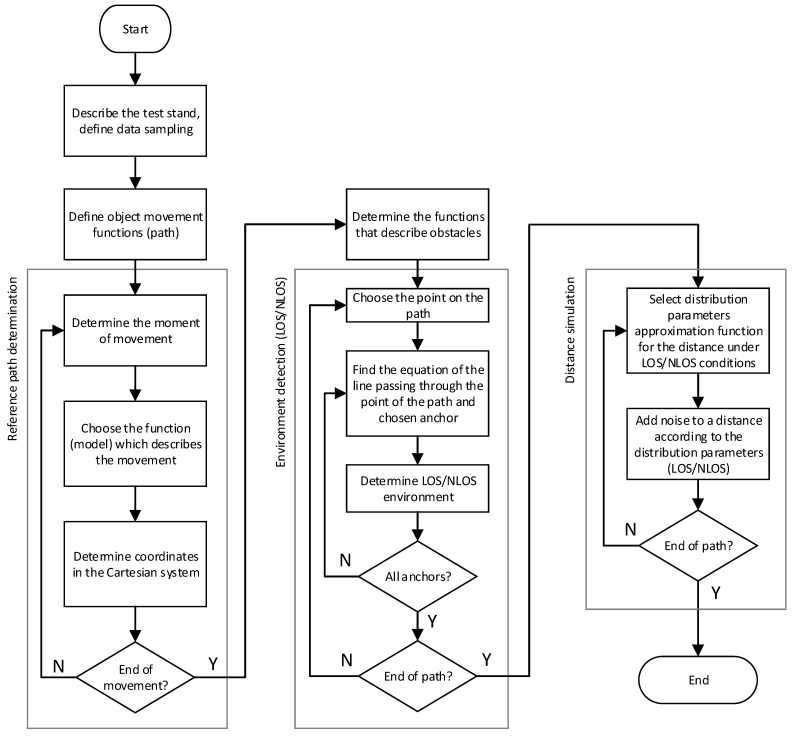
Block diagram of the simulator.

Data flow in the simulator (presented in Figure 13) is divided into three sections: determination of the reference path (reference object motion), environment detection (LOS/NLOS), and distance generation.

Step one: place (indicate) anchors in the fixed positions on the plane. The default model configuration is created automatically based on the approximated function of MBE and standard deviation. Step two: define the object’s movement with respect to time, taking into account the assumptions described in Section 3.1 and using equations of lines and arc described in Section 3.2. Step three: the reference path points are automatically calculated using the object motion function described in step two. The reference distances to anchors (defined in step one) are automatically calculated. Step four: describe obstacles using linear and nonlinear functions—described in more detail in Section 3.4. Step five: it is automatically checked whether the anchors are within LOS/NLOS conditions for each point on the path. Step six: distortion is added (see Section 3.5) to the distances (calculated in step three) using the appropriate model (some of the distances may be within LOS and others NLOS conditions, depending on the detected conditions in step five).

### 3.1. System Assumptions

**Figure 14:**
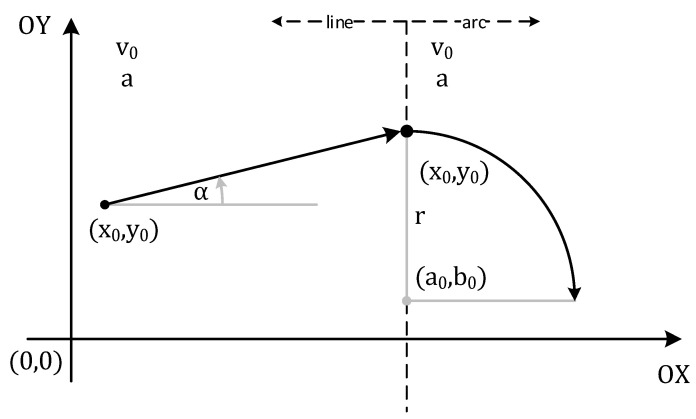
Parameters of the path in segments (for a line and arc).

The first part of the simulator environment is the description of the test scenario (i.e., the movement path of a vehicle or an object). The considerations are limited to two-dimensional spaces. The vehicle’s route (car or RC model) is approximated by straight lines and arcs (the vehicles’ turning radius is limited), as shown in Figure 14. Combining straight lines and arcs makes it possible to define various test scenarios (paths of movements) that can be observed in road traffic, especially on junctions. That movement modelling allows for more straightforward adaptation of the virtual scenario in real conditions (e.g., by a driver or an autonomous vehicle). Thus, the vehicle motion is modelled by connecting the curves together, but the variable trajectory of motion is not the only determinant of how many curves the road consists of. The division of the path (and thus the motion) into segments depends on the following parameters:

- Initial position—(x_0_, y_0_) in m;
- Initial speed—v_0_ in m/s;
- Acceleration or tangential acceleration for arc (constant or expressed as a function of time, which affects the x and y coordinate functions, further considerations include the constant acceleration)—a in m/s^2^;
- The movement direction (as the angle with the positive direction of the *X*-axis in the local coordinate system)—α in radians;
- Turning radius—r in m;
- The rotation direction in which the object rotates around an axis (clockwise or anticlockwise).

### 3.2. Motion Description

Curves (lines and arcs) describing the path are presented as a function of time, assuming constant acceleration (a = const).

In the case of lines, using the formula for the displacement in uniformly accelerated motion (4) and the trigonometric functions (5) and (6), it is possible to determine the coordinates of an object as a function of time (7) and (8):

(4) $$s=v_{0}t+\frac{1}{2}at^{2}$$

(5) $$\sin\left(\alpha \right)=\frac{y}{s}$$

(6) $$\cos\left(\alpha \right)=\frac{x}{s}$$

(7) $$x\left(t\right)=x_{0}+\cos\left(\alpha \right)\left(v_{0}t+\frac{1}{2}at^{2}\right)$$

(8) $$y\left(t\right)=y_{0}+\sin\left(\alpha \right)\left(v_{0}t+\frac{1}{2}at^{2}\right),$$

In the case of arcs, using the formula for the displacement in uniformly accelerated motion (4), the arc length Formula (9), and the parametric equation of the circle (10), and the coordinates of an object moving along an arc with a given radius as a function of time can be obtained (11) and (12):

(9) s=rα

(10) $$\{\begin{array}{c} x=a_{0}+r\cos\left(\alpha \right)\\ y=b_{0}+r\sin\left(\alpha \right) \end{array}$$

(11) $$x\left(t\right)=a_{0}+r\cos\left(\frac{v_{0}t+\frac{1}{2}at^{2}}{r}\right)$$

(12) $$y\left(t\right)=b_{0}+r\sin\left(\frac{v_{0}t+\frac{1}{2}at^{2}}{r}\right),$$

**Figure 15:**
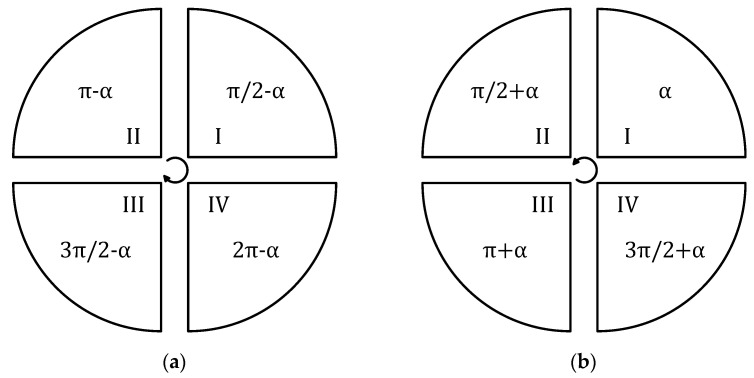
Changing the starting point of an arc (with an accuracy of 90°): (**a**) Clockwise motion and (**b**) counter clockwise motion.

Depending on the point at which the arc connects to the previous segment of the path (starting point for the current part of the path) and the direction of movement, the angle should be modified in Formulas (11) and (12). Examples of modifications with an accuracy of 90° are shown in Figure 15a for clockwise movement and Figure 15b for the counter clockwise movement.

**Figure 16:**
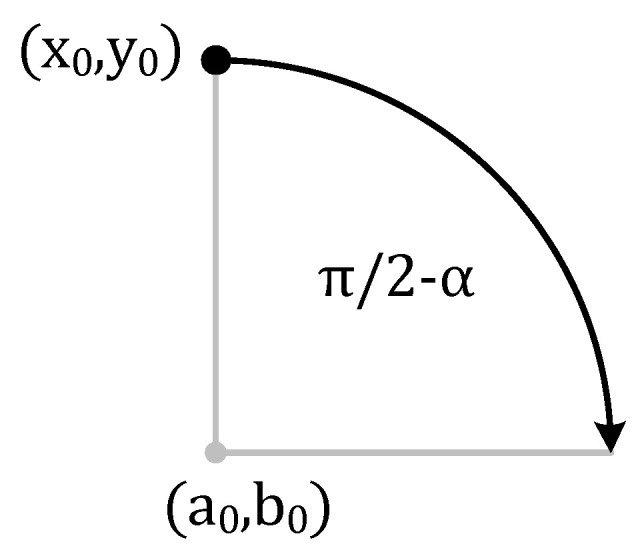
An example of a clockwise circular motion starting at 90°.

For example, for the starting point at an angle of 90° (1st quarter) and clockwise motion (see Figure 16), Equation (11) takes the form (13) and Equation (12) takes the form (14).

(13) $$x\left(t\right)=x_{0}+r\cos\left(\frac{\pi }{2}-\frac{v_{0}t+\frac{1}{2}at^{2}}{r}\right)$$

(14) $$y\left(t\right)=y_{0}-r+r\sin\left(\frac{v_{0}t+\frac{1}{2}at^{2}}{r}\right)$$

**Figure 17:**
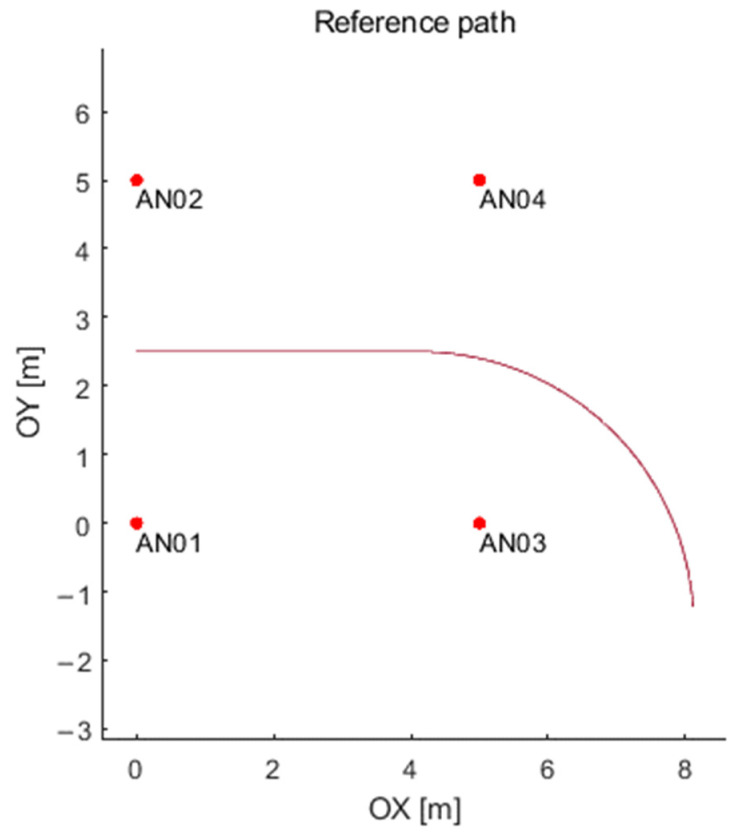
An sample reference path build of line and arc.

An example of an object path consisting of a straight line and an arc is shown in Figure 17. The number of points defining the path depends on the time chosen for a single transmission between two UWB nodes (between tag and chosen anchor).

### 3.3. UWB Distance Estimation

Euclidean distances between the tag and the anchors (15) are determined for each point on the reference path, where ($x_{A_{k}}$, $y_{A_{k}}$) are coordinates of the k-th anchor and ($x_{T},\;y_{T}$) are coordinates of the tag. The calculated distances are exact values and can be used as a ground truth for further testing of the developed algorithms.

(15) $$d_{k,i}=\sqrt{{\left(x_{A_{k}}-x_{T}\right)}^{2}+{\left(y_{A_{k}}-y_{T}\right)}^{2}}$$

Based on the calculated measures and developed models describing changes in MBE and standard deviation (σ) of the real UWB positioning system for LOS and NLOS conditions, distortion is added to the exact value (16):

(16) $$d_{k,i}^{\prime }=d_{k,i}+MBE_{d_{k,i}}+R\sigma_{d_{k,i}}$$

where $d_{k,i}$ is the distance between *k*-th anchor and *i*-th position on the path, $MBE_{d_{k,i}}$ is MBE suitable for $d_{k,i}$_,_ $\sigma_{d_{k,i}}$ is the standard deviation suitable for $d_{k,i}$, *R* is a standard normal random variable.

All determined reference distances between the tag and the anchor are modified by adding distortion according to (16) in the final phase of the simulator operation. The real UWB positioning system uses TWR (two way ranging) to obtain the distance between a tag and an anchor. If the tag is in motion, the TWR method measures the time (afterwards the time is converted into the distance) three times and presents the average time (thus, the average distance) as a result. The accuracy is good enough for static and low speed objects, where the difference in position between successive measurements is small. If the object is moving with greater speed, the TWR method itself generates lower accuracy since the following distances (measured at the device level) differ significantly. The resulting distance is an average and it refers to distance (ranges) from the past (earlier position on the path). In other words, when an object is moving, the delay (time needed for ranging) on the UWB positioning system indicates past position. The simulator has been applying the same mechanism, assuming simulator level time, e.g., 1 ms per one data exchange between a tag and an anchor (this time can be configured and adjusted to requirements). Hence, to obtain the average distance between a single anchor and a tag, an overall 3 ms are needed. If the system has four anchors, a single data packet (with distances to 4 anchors) is made available every 12 ms. On the simulator level, three distances for every 12 ms are generated based on the objects’ speed and path. The shift between distances (per single ranging) is calculated based on the current speed (configured time interval between three distances on the system level is constant, i.e., 1 ms) and defined path.

The distances are prepared in such a way that they can be treated as distances from the real system and may be applied in further processing. The mean and deviation for the measured and simulated distances are presented in Table 6.

### 3.4. Description of Obstacles

**Figure 18:**
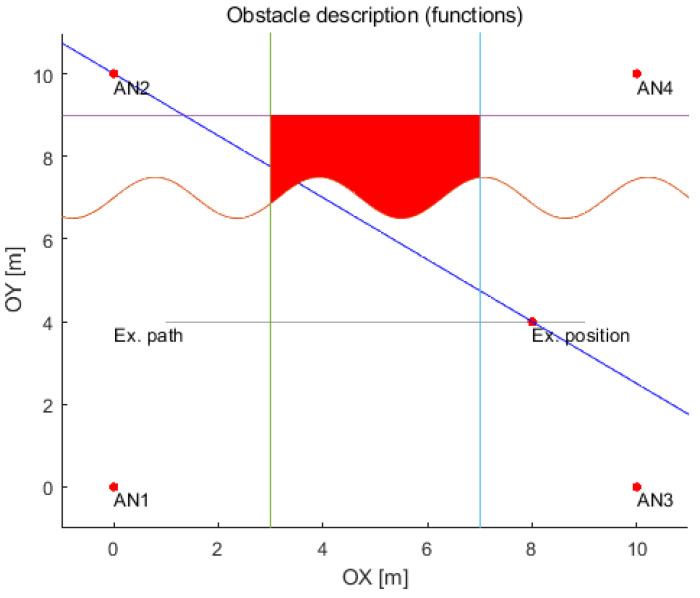
Example of the obstacle description and NLOS detection for the selected point on the path.

Introducing obstacles (not moving objects) in the simulator environment is an additional feature that gives much more possibilities of test scenarios. Other simulators do not offer LOS/NLOS detection with different ranging accuracy for them. The construction of an obstacle is based on its description with linear or nonlinear functions on the 2D plane, depending on the shape. For example, the set of functions describing one obstacle that should describe a bounded set is presented in Figure 18. Thus, the simulated transmission (distance) can be either in LOS or NLOS conditions depending on the current position of an object with respect to an anchor. Hence, each position on the track must be checked, whether the straight line passing through the tag and the chosen anchor cross the obstacle. In other words, it has been checked whether the line has at least one common point with the obstacle.

When an obstacle is described with functions, a system of inequalities is formed. It is possible to solve a system of inequalities and designate a group of possible solutions (a set of values). However, the purpose of this task is to determine whether the currently measured distance is determined under LOS or NLOS conditions. Therefore, the exact group of solutions is not needed, but only information if any solution does exist. This task can be treated as an optimisation problem, limiting the solution to examining whether there is any solution—the point where a line (passing through the tag and the chosen anchor) intersects the obstacle. If the solution is not found, it means that the distance is determined in LOS conditions, whereas if the solution is found, it means that the distance is determined in NLOS conditions.

Iterative algorithms have been applied to find the solution (to check if the connection between a tag and an anchor is under LOS or NLOS conditions). Their operation time depends on the chosen parameters (e.g., the maximum number of iterations, the minimum step value, the minimum change of the function value, etc.). The number of tests performed (detecting LOS/NLOS conditions) depends on the number of points on the path, the number of anchors, and the obstacles. Apart from the selection of the algorithm’s stop parameters, the calculations can also be paralleled by dividing the points on the path into groups to speed up the calculations. For example, for one obstacle, four anchors, and one tag, the simulation lasts about seven hours (on a PC with a six-core processor) for about 10,000 points on the reference path (i.e., making 1×4×1×10,000=40,000 checks). The time of simulation increases linearly with the number of checks performed (e.g., for two obstacles, four anchors, and one tag and for 10,000 points on the path the simulator performs 2×4×1×10,000=80,000 checks and it lasts about 14 h). The time of simulation can be reduced by using grid mapping—it divides the test stand into cells (resulting in fewer checks). It is possible to generate an occupancy grid with adjustable cell size. It may reduce the simulation time significantly.

### 3.5. Simulation of Distances

**Figure 19:**
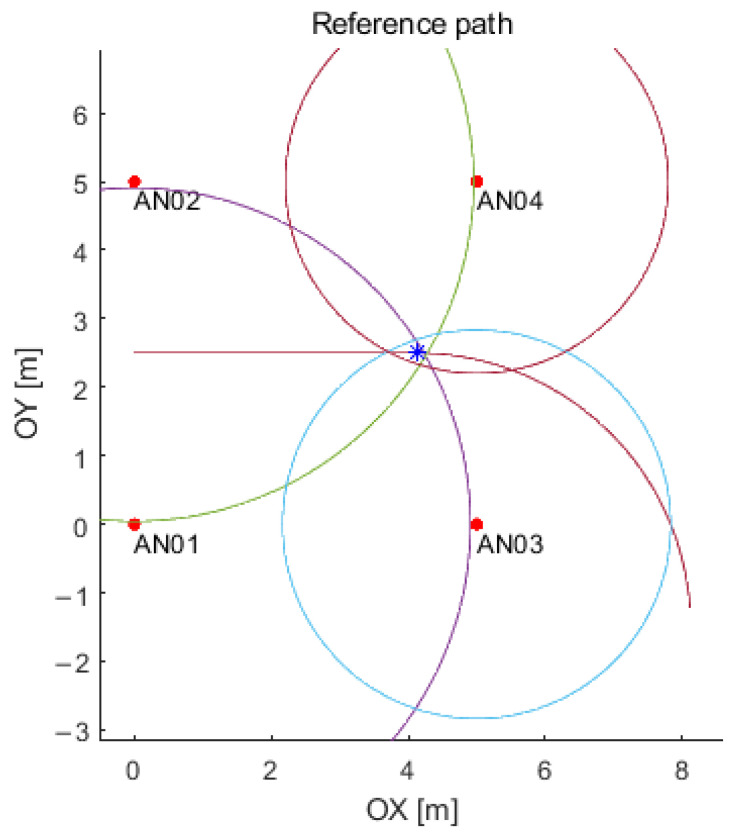
Example of the distance simulation for a single point (i.e., a tag, blue asterisk) on the path.

It is possible to generate data from the UWB system with the use of the designed and created simulator in laboratory conditions based on: the data of the error values; standard deviations (for both environments); the description of the environment (with or without obstacles); and a path shape (see Figure 19). The red line shows the reference track and the circles represent distances between each anchor and a sample point on the track (tag). The obtained distances allow to determine the object’s position in the further stages of data analysis and to correct them.

**Figure 20:**
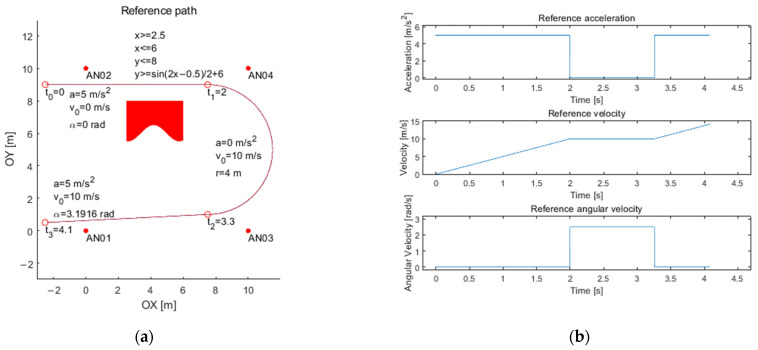
Example of (**a**) path with parameters and (**b**) motion description.

**Figure 21:**
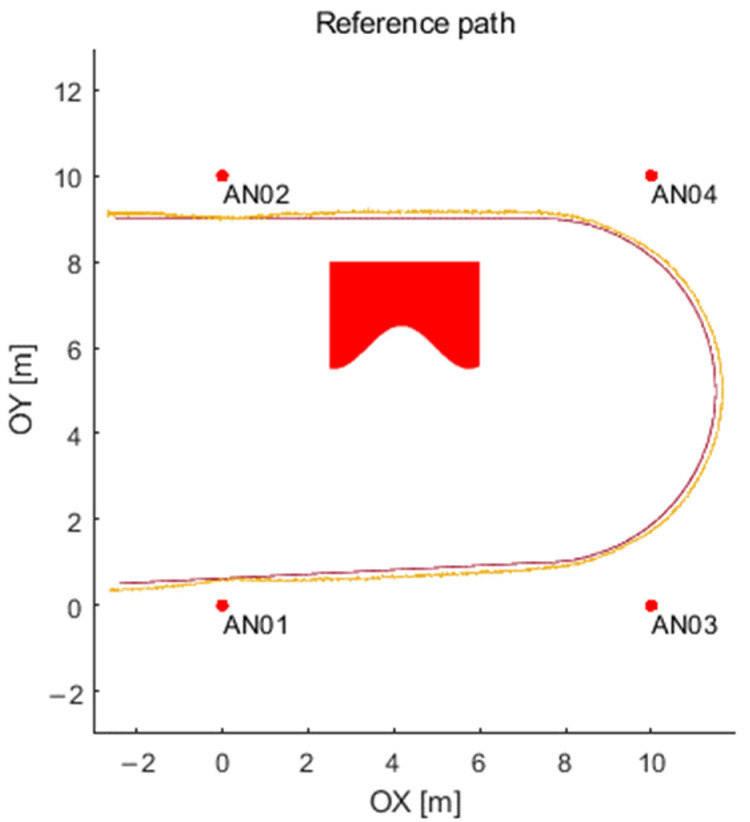
Path calculated (by trilateration algorithm) using generated distances.

An exemplary path consisting of three segments with parameters is presented in Figure 20. The exemplary path contains three segments (two linear segments and an arc). The first linear segment is defined by a timeslot from t_0_ = 0 s up to t_1_ = 2 s, start position of x_0_ = −2.5 m, y_0_ = 9 m, an acceleration 5 m/s^2^, the initial velocity of 0 m/s and the direction of the movement (as the angle with the positive direction of the *X*-axis) equals 0 rad. The second segment (an arc) is defined by the timeslot from t_1_ = 2 s up to t_2_ = t_1_ + 1.2566 s, the initial position is the last position from the previous segment, the acceleration equals 0 m/s^2^, the initial velocity equals 10 m/s (it is a continuous value), and the arc radius is 4 m. The third segment is defined by the line within timeslot from t_2_ = 3.2566 s up to t_3_ = t_2_ + 0.83 s, the initial position is the last position from the previous segment (the arc segment), the acceleration equals 5 m/s^2^, the initial velocity 10 m/s, and the direction of the movement equals 3.1916 rad. The distances for the trilateration process are available for every data packet (12 times system interval). The distance between the tag to the four nearest anchors and the time shift of an object is included. Simulator output, i.e., a path calculated (by trilateration algorithm) using generated distances, can be seen in Figure 21. The data obtained may be further utilized for various purposes. Still, the most important is to have a reference path and a real UWB system simulator described by the proposed model.

## 4. Conclusions

The designed and created UWB 2D positioning system simulator can be applied to simulate the movement of objects such as AGVs, vehicles, cars, and RC models and has numerous advantages. It allows for defining a customised path of an object, taking into account initial velocity, acceleration, and type of path (line or arc). A number of the required positioning devices (anchors) that can be placed on the plane are flexible. The simulator has a unique feature in the form of obstacles description. The user defines the number of obstacles, and they can be placed in any place on the plane. It leads to another great advantage of the simulator that is taking into consideration LOS and NLOS conditions.

There are no reliable UWB simulators that can be applied for objects in motion, taking into account the changing accuracy of the system along with the distance and LOS/NLOS conditions. The presented simulator has been designed and its properties have a strong relationship to the real UWB system. Such an approach allows for further data analysis and it does not require repetitive test scenarios in the field. The presented simulator allows to emulate the real positioning system for further evaluation of various algorithms of raw data processing or trilateration. The presented analysis of data from the UWB system shows the error variability along with the distance and the impact of the obstacle on the measured distances. For this reason, the simulator uses set of functions to generate distances and is equipped with the possibility to add obstacles and detection of LOS/NLOS conditions.

The construction of the simulator based on modelled changes in distribution parameters allows for quick adaptation of the simulator to another UWB system (another model or produced by another manufacturer). The simulation time can be a limitation because of the LOS/NLOS detection module (time of simulation increases with a number of reference points and obstacles).

Conducting multiple tests in real conditions (in the field) is time consuming. The simulator makes it easy to describe various test scenarios and generate UWB data according to a defined path. The presented description (of obstacle and motion) allows for a relatively simple transfer of a virtual (simulated) test scenario to a real test stand which simplifies the comparison of results. A particular advantage of the simulator is the accurate ground truth (i.e., exact distances between nodes) which is necessary when preparing algorithms that should increase the accuracy of the determined position and which is extremely difficult to find (accuracy of the millimetres order and high sampling frequency).

## Figures and Tables

**Table 1:** Reference distances.

Distances [cm]	50; 100; 150; 200; 250; 300; 350; 400; 450; 500; 550; 600; 650; 700; 750; 800; 850; 900; 950; 1000; 1100; 1200; 1300; 1400; 1500; 1600; 1700; 1800; 1900; 2000
Measurements [samples/distance]	10,000
Overall LOS/NLOS [samples]	600,000 (2 × 300,000)

**Table 2:** Statistical measures of the distance under LOS conditions.

Reference [cm] d_ref_	Mean [cm] d_mean_	Median [cm] d_med_	STD [cm] d_std_	Min [cm] d_min_	Max [cm] d_max_	|d_mean_ − d_med_| [cm]
50	52.0	52	1.8	45	59	0.0
100	108.5	109	2.4	94	118	0.5
150	157.7	158	2.4	147	166	0.3
200	209.2	209	2.3	200	218	0.2
250	264.5	265	2.1	254	273	0.5
300	305.2	305	2.1	299	318	0.2
350	362.2	362	2.5	353	371	0.2
400	407.3	407	1.9	401	414	0.3
450	459.0	459	1.5	453	466	0.0
500	513.5	513	2.2	503	522	0.5
550	565.6	566	2.5	554	572	0.4
600	614.2	614	1.9	607	621	0.2
650	660.5	661	1.4	653	665	0.5
700	707.0	707	1.4	702	713	0.0
750	763.4	763	1.8	758	770	0.4
800	816.1	816	2.0	807	823	0.1
850	865.6	866	2.1	856	875	0.4
900	916.1	916	2.6	908	925	0.1
950	967.3	967	2.2	959	976	0.3
1000	1018.5	1019	2.4	1010	1026	0.5
1100	1117.3	1117	2.0	1111	1124	0.3
1200	1210.7	1211	1.4	1205	1216	0.3
1300	1311.2	1311	1.0	1308	1315	0.2
1400	1407.6	1408	1.5	1402	1415	0.4
1500	1519.8	1520	1.6	1513	1525	0.2
1600	1621.3	1621	1.6	1616	1627	0.3
1700	1716.4	1716	1.8	1710	1724	0.4
1800	1820.1	1820	1.5	1814	1826	0.1
1900	1919.3	1919	1.5	1913	1925	0.3
2000	2015.6	2015	1.7	2010	2021	0.6

**Table 3:** Statistical measures of the accuracy (distance error) under LOS conditions.

Reference [cm] d_ref_	*MBE* [cm] d_mbe_	*RMSE* [cm] d_rmse_	STD [cm] d_std_	ε_min_ [cm]	ε_max_ [cm]	|ε|_min_ [cm]	|ε|_max_ [cm]
50	2.0	2.7	1.8	−5	9	0	9
100	8.5	8.9	2.4	−6	18	0	18
150	7.7	8.1	2.4	−3	16	0	16
200	9.2	9.5	2.3	0	18	0	18
250	14.5	14.7	2.1	4	23	4	23
300	5.2	5.6	2.1	−1	18	0	18
350	12.2	12.4	2.5	3	21	3	21
400	7.3	7.5	1.9	1	14	1	14
450	9.0	9.1	1.5	3	16	3	16
500	13.5	13.6	2.2	3	22	3	22
550	15.6	15.8	2.5	4	22	4	22
600	14.2	14.4	1.9	7	21	7	21
650	10.5	10.6	1.4	3	15	3	15
700	7.0	7.2	1.4	2	13	2	13
750	13.4	13.5	1.8	8	20	8	20
800	16.1	16.2	2.0	7	23	7	23
850	15.6	15.8	2.1	6	25	6	25
900	16.1	16.3	2.6	8	25	8	25
950	17.3	17.4	2.2	9	26	9	26
1000	18.5	18.7	2.4	10	26	10	26
1100	17.3	17.4	2.0	11	24	11	24
1200	10.7	10.8	1.4	5	16	5	16
1300	11.2	11.2	1.0	8	15	8	15
1400	7.6	7.8	1.5	2	15	2	15
1500	19.8	19.8	1.6	13	25	13	25
1600	21.3	21.4	1.6	16	27	16	27
1700	16.4	16.5	1.8	10	24	10	24
1800	20.1	20.2	1.5	14	26	14	26
1900	19.3	19.4	1.5	13	25	13	25
2000	15.6	15.6	1.7	10	21	10	21

**Table 4:** Statistical measures of the distance under NLOS conditions.

Reference [cm] d_ref_	Mean [cm] d_mean_	Median [cm] d_med_	STD [cm] d_std_	Min [cm] d_min_	Max [cm] d_max_	|d_mean_ − d_med_| [cm]
50	99.8	100	1.1	96	107	0.2
100	179.4	181	7.9	120	186	1.6
150	225.6	228	5.6	190	234	2.4
200	227.4	230	17.6	196	279	2.6
250	262.3	258	10.9	251	323	4.3
300	309.6	310	1.4	305	315	0.4
350	433.3	433	3.1	375	439	0.3
400	408.4	408	1.4	403	414	0.4
450	511.2	511	10.6	476	538	0.2
500	548.7	547	10.5	518	582	1.7
550	635.2	638	8.6	591	645	2.8
600	602.9	603	1.5	597	609	0.1
650	650.8	651	1.3	646	657	0.2
700	769.5	770	8.9	720	784	0.5
750	824.3	824	1.3	808	829	0.3
800	803.2	803	1.5	798	810	0.2
850	852.3	852	4.5	845	934	0.3
900	898.6	895	8.9	890	940	3.6
950	955.0	955	2.5	950	999	0.0
1000	1009.9	1010	1.9	1003	1026	0.1
1100	1163.0	1163	2.7	1136	1169	0.0
1200	1207.7	1208	1.6	1201	1213	0.3
1300	1348.2	1349	9.2	1319	1372	0.8
1400	1403.6	1399	10.5	1390	1471	4.6
1500	1498.4	1498	1.2	1494	1503	0.4
1600	1660.9	1661	9.8	1626	1677	0.1
1700	1757.3	1757	1.9	1751	1764	0.3
1800	1835.5	1836	14.2	1800	1873	0.5
1900	1901.3	1901	1.4	1896	1907	0.3
2000	2014.9	2015	18.0	1993	2077	0.1

**Table 5:** Statistical measures of accuracy (distance error) under NLOS conditions.

Reference [cm] d_ref_	*MBE* [cm] d_mbe_	*RMSE* [cm] d_rmse_	STD [cm] d_std_	ε_min_ [cm]	ε_max_ [cm]	|ε|_min_ [cm]	|ε|_max_ [cm]
50	49.8	49.8	1.1	46	57	46	57
100	79.4	79.8	7.9	20	86	20	86
150	75.6	75.8	5.6	40	84	40	84
200	27.4	32.6	17.6	−4	79	0	79
250	12.3	16.4	10.9	1	73	1	73
300	9.6	9.8	1.4	5	15	5	15
350	83.3	83.3	3.1	25	89	25	89
400	8.4	8.5	1.4	3	14	3	14
450	61.2	62.1	10.6	26	88	26	88
500	48.7	49.8	10.5	18	82	18	82
550	85.2	85.6	8.6	41	95	41	95
600	2.9	3.3	1.5	−3	9	0	9
650	0.8	1.5	1.3	−4	7	0	7
700	69.5	70.1	8.9	20	84	20	84
750	74.3	74.3	1.3	58	79	58	79
800	3.2	3.5	1.5	−2	10	0	10
850	2.3	5.0	4.5	−5	84	0	84
900	−1.4	9.0	8.9	−10	40	0	40
950	5.0	5.5	2.5	0	49	0	49
1000	9.9	10.1	1.9	3	26	3	26
1100	63.0	63.1	2.7	36	69	36	69
1200	7.7	7.9	1.6	1	13	1	13
1300	48.2	49.1	9.2	19	72	19	72
1400	3.6	11.1	10.5	−10	71	0	71
1500	−1.6	2.0	1.2	−6	3	0	6
1600	60.9	61.7	9.8	26	77	26	77
1700	57.3	57.3	1.9	51	64	51	64
1800	35.5	38.3	14.2	0	73	0	73
1900	1.3	1.9	1.4	−4	7	0	7
2000	14.9	23.3	18.0	−7	77	0	77

**Table 6:** Comparison of real data with simulated data.

Reference [cm] d_ref_	Measured Distances [cm]		Simulated Distances [cm]		Difference	
	d_mean_	d_std_	d_mean_	d_std_	d_mean_	d_std_
LOS						
1	108.5	2.4	108.5	2.3	0.0%	3.1%
2	209.2	2.3	209.2	2.3	0.0%	0.6%
3	305.2	2.1	307.0	2.1	0.6%	0.8%
5	513.5	2.2	513.1	2.3	0.1%	5.7%
7	707.0	1.4	707.0	1.4	0.0%	2.3%
10	1018.5	2.4	1017.8	2.4	0.1%	2.2%
13	1311.2	1.0	1309.8	1.0	0.1%	1.2%
16	1621.3	1.6	1621.3	1.7	0.0%	3.6%
19	1919.3	1.5	1919.3	1.5	0.0%	0.0%
NLOS						
1	179.4	7.9	182.1	7.9	1.5%	0.8%
2	227.4	17.6	235.6	17.4	3.6%	0.8%
3	309.6	1.4	309.7	1.4	0.0%	0.8%
5	548.7	10.5	548.7	11.1	0.0%	5.8%
7	769.5	8.9	770.5	8.9	0.1%	0.8%
10	1009.9	1.9	1016.8	1.9	0.7%	0.8%
13	1348.2	9.2	1348.3	9.1	0.0%	0.8%
16	1660.9	9.8	1658.5	9.7	0.1%	0.8%
19	1901.3	1.4	1901.3	1.4	0.0%	0.8%

## Data Availability

The data presented in this study are available on request from the corresponding author.
